# Connectivity Analysis during Rubber Hand Illusion—A Pilot TMS-EEG Study in a Patient with SCI

**DOI:** 10.1155/2021/6695530

**Published:** 2021-02-08

**Authors:** Vanessa N. Frey, Aljoscha Thomschewski, Patrick B. Langthaler, Alexander B. Kunz, Yvonne Höller, Eugen Trinka, Raffaele Nardone

**Affiliations:** ^1^Department of Neurology, Christian Doppler University Hospital, Paracelsus Medical University and Centre for Cognitive Neuroscience, Austria; ^2^Spinal Cord Injury and Tissue Regeneration Center, Paracelsus Medical University, Salzburg, Austria; ^3^Department of Mathematics, Paris Lodron University, Salzburg, Austria; ^4^Karl Landsteiner Institute for Neurorehabilitation and Space Neurology, Salzburg, Austria; ^5^Department of Psychology, University of Akureyri, Akureyri, Iceland; ^6^Department of Neurology, Tappeiner Hospital, Meran, Italy

## Abstract

**Background:**

Bodily self-perception is an important concept for several neurological disorders, including spinal cord injury (SCI). Changing one's bodily self-perception, e.g., via rubber hand illusion (RHI), induces alterations of bottom-up and top-down pathways and with this the connectivity between involved brain areas. We aim to examine whether (1) this process can be manipulated by changing cortical excitability, (2) connectivity between relevant brain areas differ when the RHI cannot be evoked, and (3) how this projection differs in a patient with SCI.

**Method:**

We applied RHI and facilitatory theta burst stimulation (TBS) on the right primary somatosensory cortex (S1) of 18 healthy participants and one patient with incomplete, cervical SCI. During RHI, we recorded high-density electroencephalography (HD-EEG) and extracted directed and nondirected connectivity measures.

**Results:**

There is no difference in connectivity between sham and real TBS or in the effectivity of RHI. We observed a higher laterality in the patient, i.e., higher connectivity of the right and lower of the left hemisphere. Besides this, connectivity patterns do not differ between healthy participants and the patient.

**Conclusion:**

This connectivity pattern might represent a neuroplastic response in the attempt to overcome the functional impairment of the patient resulting in a similar overall connectivity pattern to the healthy participants, yet with a higher sensitivity towards RHI and a higher laterality. The cortico-cortical communication was not altered depending on whether the illusion was provoked or not; hence, the perceptory illusion could not be observed in the EEG analysis.

## 1. Introduction

Humans understand an object rather as a whole structure, not as the sum of its single parts [1, 2]. This idea was rekindled in the early 20^th^ century by German and Austrian psychologists (Kurt Koffka, Wolfgang Köhler, and Max Wertheimer) and embedded in the emerging “Gestalt Psychology.” This phenomenon builds the basis for illusions resulting from visual, tactile, or auditory conflicts. One illusion approaching the visuo-tactile perception and the feeling of embodiment is the rubber hand illusion (RHI) [3]. In order to evoke this illusion, the own real hand is out of view and stroked simultaneously with a fake rubber hand that lies right in front of the participant. The stimulation is being felt on the own hand but is seen on the rubber hand; hence, the tactile information conflicts with the visual perception that does not correspond to the learned association from experience. According to Barrett and colleagues, this conflict produces a prediction error that needs to be solved [4]. The direction of information processing involved can be categorized into two paths: bottom-up and top-down. Bottom-up describes the way how sensory information we adopt unconsciously is forwarded from hierarchically lower brain areas, the ones that receive the sensory input, to the hierarchically higher brain areas [5]. The top-down process manipulates the received information based on preexisting neural circuits that are built on prior experience and, thus, give rise to expectations and specific interpretations of the information given. During the illusion, there is a need to match the contradictory information about the prediction based on visual perception and the actual incoming sensory information. The manipulated information is then being fed back to the hierarchically lower brain areas via top-down projection where the modified information about the sensory input is adapted. In contrast to bottom-up, top-down signals are consciously accessible [6]. Still, it is a contradictory issue to what extend the RHI is influenced by bottom-up or top-down projection, and the factors which determine whether a body part becomes part of the illusion or not are still not clear [5]. Some studies point out that the visuotactile component is the driving force of the illusion [7], i.e., a bottom-up projection. In contrast, Tsakiris and colleagues state that multisensory input is not sufficient for the feeling of ownership [8, 9]. For a successful illusion, top-down matching is also necessary, providing the subjective experience of one's own body representation [10]. This recalculation of self-perception and feeling of embodiment might be used as a possible therapeutic approach after deafferentation, as observed in patients with spinal cord injury (SCI). Lenggenhager and colleagues demonstrated that patients with somatosensory impairment in their fingers show improvement or even regain of the tactile sensation of previously numb fingers after treatment with RHI [11]. Pazzaglia and colleagues reported similar results including a reduction of pain sensation in the patient in some fingers after the RHI [12].

**Figure 1:**
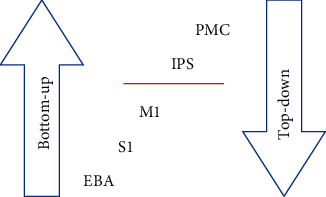
Hierarchical order of the brain areas relevant in this work. EBA: extrastriate body area; S1: primary somatosensory cortex; M1: primary motor cortex; IPS: intraparietal sulcus; PMC: premotor cortex.

Recent studies conclude that the multisensory signals induced by the RHI are being processed by a range of brain areas of the frontal, occipital, and parietal lobes [5, 13–15]: primary somatosensory cortex (S1), primary motor cortex (M1), intraparietal sulcus (IPS), premotor cortex (PMC), and extrastriate body area (EBA). These brain areas are involved in ascending and descending information forwarding and processing, creating a hierarchical structure [16]. This plays an important role in the processing of sensory information and with this the induction of the RHI. While the brain areas receiving the sensory input (S1, M1, EBA) are regarded as hierarchically lower areas, the brain areas such as PMC and IPS that are further processing the information are attributed to a hierarchically higher position [5, 16], as shown in Figure 1.

It is important to gain a better understanding of the connectivity during RHI and its possible manipulation of the bottom-up and top-down processes. This could trigger new therapeutic approaches, which influence neuroplastic changes in patients with SCI and improve their sensory perception, as demonstrated in our earlier behavioral study [17]. In order to test these pathways, modulation of processing capacities would allow for establishing causal relationships. Comparing patients with SCI with healthy participants requires a large sample size due to high variability in individual injury outcomes. Still, functional connectivity analysis might be worthwhile on an individual level to uncover structural changes and adapt personalized therapy.

In this study, we combined the RHI with facilitating TMS of the S1 and analyzed functional connectivity between the relevant brain areas in healthy participants as well as in one patient with SCI. Our aim was to investigate the effects of repetitive transcranial magnetic stimulation (rTMS) on bottom-up and top-down projections in healthy subjects and a patient with SCI. RTMS allows both for facilitating and inhibiting neural activity [18]. We decided to apply intermittent theta burst stimulation (iTBS), a well-established rTMS protocol that has been shown to increase cortical excitability [18]. Additionally, we considered participants in whom the RHI could not be provoked, since we hypothesized that there would be a weaker connectivity from the hierarchically higher to hierarchically lower areas. The method with which we investigated communication between relevant brain areas is high-density EEG, which allows to disentangle information flow at a high time resolution.

## 2. Methods

### 2.1. Participants

Twenty-one healthy participants were recruited, of which one terminated the study for personal reasons. Two further participants had to be excluded from data analysis as the quality of the EEG recording was not sufficient. In the final sample, 10 men and 8 women, with a mean age of 30 years (SD 9.2 years) were included. In three healthy participants, the illusion could not be elicited. Three patients with SCI were recruited, of which one had to be excluded as the TMS intensity had to be decreased to a minimum due to a hypersensitivity of the patient. In another patient, no MEPs could be elicited by the TMS; hence, the patient had to be excluded in the first session. One 64-year-old patient with an incomplete, nontraumatic SCI (AIS C) at a level sub-C4 was included in the study. The injury was caused by a Staphylococcus aureus sepsis, 6 months earlier.

### 2.2. Ethics

The study was approved by the local ethics committee (Local Ethics Committee Salzburg; 415-E/2085/4-2016), and all participants signed an informed consent form.

### 2.3. Study Design

**Figure 2:**
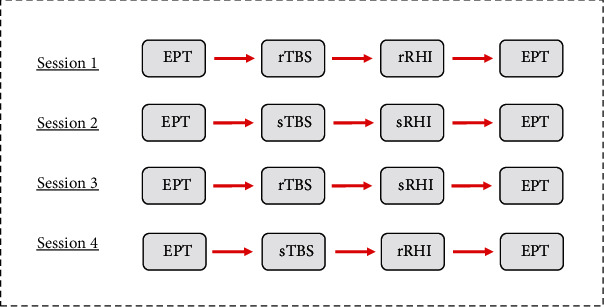
Four sessions with one week interval. EPT: electrical perception test; rTBS: real theta burst stimulation; sTBS: sham theta burst stimulation; rRHI: real rubber hand illusion; sRHI: sham rubber hand illusion.

The experiment consisted of four sessions, with intervals of at least one week in-between to avoid carry-over effects. In every session, the participants received rTMS stimulation, either real or sham, followed by a RHI, either sham or real during which HD-EEG was recorded (Figure 2). For the analysis at hand, only the data of the real RHI conditions was used.

### 2.4. Rubber Hand Illusion

The participants received visuotactile stimulation by timely synchronous brushing of the own hidden hand and a visible rubber hand that was placed in an anatomically reasonable position. The stimulation was performed for two minutes with a frequency of approximately 1 Hz, while in the sham condition, the real hand and the rubber hand were stimulated asynchronously with a delay of approx. 500 ms. During a successful RHI, the participant feels the brush touching his hand, while seeing the rubber hand being touched, causing the feeling of ownership of the fake hand. The effectivity of the illusion was tested via a standardized 9 items questionnaire filled out by the participant right after the illusion of every session. If at least 8 out of 9 items were rated with the lowest score in all four sessions, the illusion was regarded as not being successfully induced.

The procedure of the RHI and the questionnaires were adopted from Botvinick and Cohens [3].

### 2.5. Theta Burst Stimulation

We used a Power Mag research device by Mag &More GmbH and the software rTMS interface to program the TMS protocol. To test the individual resting motor threshold (RMT) and detect the motor hot spot of the first dorsal interosseous (FDI) of the right hemisphere, single TMS pulses were applied following the procedure described by Rossini et al. [19].

We used iTBS to reach the maximum facilitating effect, consisting in a higher frequency delivered as three pulses with 50 Hz, given in ten trains for 2 seconds following 8 seconds of break. This is repeated 20 times, summing up to 600 pulses in 200 seconds [20]. The intensity was reduced to 80% of RMT, and the iTBS was applied at the primary somatosensory cortex (S1) contralateral to the left FDI (2 cm posterior shift from the motor hotspot). In the sham condition (sTBS), the same stimulation protocol was applied as in the real condition (rTBS), yet the TMS coil was flipped away from the skull at 90 degrees.

### 2.6. EEG Recording and Preprocessing

EEG was recorded with 256-channel HydroCel geodesic sensor nets and a GES 400 amplifier (Koninklijke Philips N.V., Amsterdam: Netherlands). The data was recorded at 1000 Hz sampling rate using Philips' NetStation 4.5.6 software. Adhering to the proposed guidelines [21–23], impedances were kept below 75 k*Ω*. EEG data was first filtered between 0.1 Hz and 80 Hz using an FIR Bandpass filter. In addition, a 50 Hz notch filter was applied to account for line noise corrupting the data. Next, the data was segmented for each experimental condition into equally sized segments of 500 ms.

Artifact rejection was performed on these segments using the artifact rejection tool within NetStation. Segments that were too much corrupted by artifacts were excluded from further analyses.

### 2.7. EEG Connectivity Analysis

Within Matlab [24], EEG channels were averaged according to 10 predefined regions over both hemispheres (see Supplementary Figure S1 for brain regions and electrodes). Autoregressive models were calculated for each segment using the mvfreqz.m and mvar.m function implemented within the BioSig toolbox [23]. The multivariate autoregressive models were calculated for all region × region combinations and for the three frequency bands of interest (alpha: 7-13 Hz, low gamma: 40-50 Hz, and high gamma: 51-79 Hz) with a model order 15, chosen in order to adhere to the proposed ratio of 3 : 1 between given samples and the number of estimates [25]. From the multivariate autoregressive model, we derived two measures of interaction: ordinary coherence (COH), which is an undirected measure considering the real part of the complex-valued coherence [23], and the full frequency directed transfer function (ffDTF), a directed measure of interaction normalized with respect to all the frequencies in the predefined frequency interval [26].

### 2.8. Statistics

We compared the sTBS with the rTBS condition and subjects in whom the RHI could be elicited (RHI) with those in whom it did not work (noRHI). For both comparisons, we took the mean overall segments of one condition within each person for every region × region × frequency combination. We then conducted permutation tests as follows: for the RHI vs. noRHI comparison, we computed absolute values of *t*-statistics for the original observations and for 10.000 permutation samples. Since the sample size was too small to achieve adequate power when correcting the *p* values for such a high number of tests, we chose to conduct global tests and only investigate further if those were significant. From this, we computed an individual *p* value for each set of variables, which we then combined using the Nonparametric Combination Methodology (NPC) described by Pesarin and Salmaso [27]. As a combining function, we used Fisher's combining function. For the rTBS vs. sTBS comparison, we computed the difference of values between rTBS and sTBS within each subject. We then computed the absolute value of the sum of these values divided by its standard deviation for the original data and 10.000 random permutation samples that were generated by randomly varying the signs of the differences [27]. Statistical analysis was conducted using the software package R [28]. The distribution of the patient's data is shown within the data of the healthy control group as percentiles.

## 3. Results

The global *p* values show no difference of directed (ffDTF) and nondirected (COH) connectivity between real and sham TBS within the participants. Neither are there significant differences between the participants in whom the RHI could be elicited and the ones in whom it was not (Table 1).

**Figure 3:**
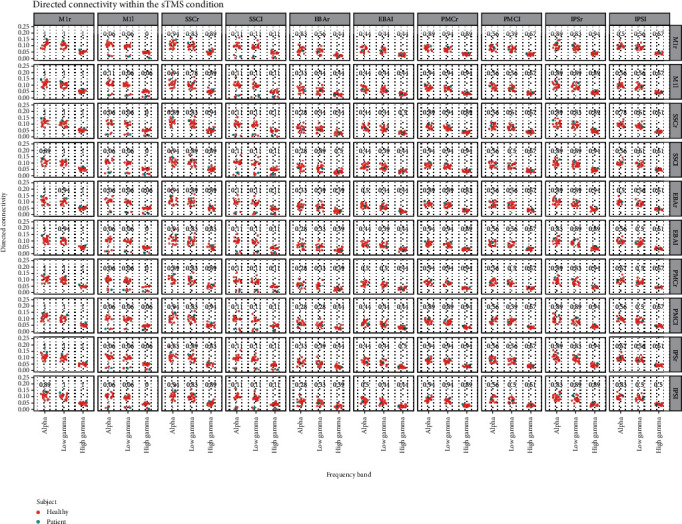
Directed connectivity (ffDTF) after sham transcranial magnetic stimulation. The values show the percentile of the patients' data within the data of the control group (from 0 to 1). M1: primary motor cortex; S1: primary somatosensory cortex; EBA: extrastriate body area; PMC: premotor cortex; IPS: intraparietal sulcus; r: right; l: left.

Looking at the distribution of the patient's data within the data of the healthy control group, we observed rather distinctive trends. After sTBS, the patients' connectivity output is higher in the brain regions of the right hemisphere: M1, S1, PMC, and IPS, compared to the healthy control group, while the left M1 and S1 show lower connectivity (for values see Figure 3). The directed connectivity after rTBS shows fewer differences between the patient and the healthy control group: The left and especially right EBA and IPS left show lower directed connectivity, while the left and right M1 show higher connectivity.

The coherence analysis does not reveal any remarkable differences after sTBS. Yet after rTBS, the PMC and M1 show higher connectivity in the patient compared to the control group (for values see Supplementary Figures S2–S5).

## 4. Discussion

In this pilot study, we investigated the effect of RHI and repetitive TMS in healthy participants and one patient with cervical SCI on HD-EEG. In our earlier work [17], we demonstrated a behavioral suppression of the RHI after facilitating TBS stimulation over S1. Additionally, we found an increase in tactile sensation in the patient after applying the RHI. In the presented work, we examined connectivity patterns during RHI paired with either real or sham TBS.. While interpreting the data we just presented, it should be kept in mind that the rTBS condition might decrease the effectivity of the RHI, possibly by strengthening the bottom-up connection.

Referring to the results discussed so far, the group of healthy participants in whom the RHI could not be elicited does not stand out. This suggests that the processes taking place during visuotactile stimulation are independent of the final outcome of body perception. The intensity and pattern of directed and nondirected connectivity that we recorded do not correlate with the final perception of whether the rubber hand is being integrated or not. It might rather reflect the mechanisms taking place when receiving multisensory inputs that generate contradictory information. Hence, the connectivity observed might reflect the mechanism of processing a prediction error, without influencing the bodily self-perception yet. This matches with the behavioral data of our earlier work [17] as the sham RHI conditions revealed a stronger effect than expected. It seems that the rubber hand is integrated well and quickly into the own body perception, while it might actually be the asynchronous sham stimulation that really causes struggle in the feeling of embodiment. This effect was observed by Karabanov and colleagues, as there was a sensorimotor conflict only if the rubber hand illusion was not successfully elicited [29]. Zeller and colleagues observed a stronger response in M1 and S1 during the nonillusion condition compared to the illusion condition [30]. This phenomenon can be compared with observations made during motor imagery tasks. Neuronal mechanisms activated during motor imagination are very similar to those being responsible for preparing, programming, and conducting the movement [31, 32]. Processes during RHI comprise observing and imagining of movements, including assimilation of proprioceptive and sensorimotor information. It should be noted that it is not clear if the analysis we conducted is of sufficient sensitivity to detect the difference between an illusion being elicited or not.

The fact that there is no significant difference in connectivity between the sham and real TBS stimulation is rather surprising. On the one hand, there might be an overlapping effect of the RHI over the TMS regarding communication of the brain areas. On the other hand, TMS does have an influence on the behavioral data of the study [17]. Hence, processing the multisensory information during the RHI shows a greater effect regarding connectivity than a change of cortical excitability. For future studies, it would be interesting to apply inhibiting, instead of enhancing, TMS to S1, e.g., continuous TBS. Suppressing S1 during RHI might strengthen the top-down projection and with this increase the effectivity of the illusion.

Even though the patient shows similar patterns of connectivity as the healthy participants, some differences were found when plotting the data sets in percentiles. The patient shows lower connectivity in the left hemisphere and higher connectivity in the right hemisphere, compared with the data pool of all participants. Interestingly, lateralization has been also observed in a motor imagery study by Athanasiou and colleagues [33]. They found higher outflow from the right and higher inflow in the left cingulate motor areas during visual-motor imagery tasks of the upper extremities and walking. In patients with SCI, this effect retained, yet to a lower extent than in healthy participants, in contrast to our data obtained during RHI. Lateralization can be observed mainly after sTBS, increasing the effectivity of the RHI according to behavioral data [17]. The increased laterality in the patient points to a higher effectivity of the RHI compared to the healthy control group.

A possible explanation for the lower connectivity of the left hemisphere might be that the right hand is not included in the RHI. It is placed on the lap and does not receive any sensory input, while the attention lies on the left hand. For these reasons, the right hand might have been rather neglected provoking a decrease in connectivity within the left hemisphere.

However, the overall difference between the patient and the healthy control group seems to be small, pointing to a possible adjustment of connectivity and communication output after deafferentation. It was shown before that during movement preparation, there is a possible compensation in patients with SCI regarding the efficiency [34], representing the effectiveness of communication between two regions [35]. The authors stated that global efficiency in patients with SCI might be the same as in a healthy control group, even though local efficiency is increased in their study [30] and decreased in another study [36]. In any case, for making clear statements and comparisons between healthy participants and patients with SCI, higher sample sizes are necessary. Further studies revealed that changes in functional brain connectivity in patients with SCI can vary depending on their injury's outcome, as AIS score, and might even serve as a predictor for motor recovery [37–39]. Hence, even within a patient group, there might be high inhomogeneity in injury outcome influencing connectivity patterns. This makes it necessary to investigate larger patient groups, either divided into subgroups or implementing limited inclusion criteria. Comparing a group of healthy participants with one patient with SCI does not give information about the correlation of functional connectivity and single characteristics of the injury outcome. Yet, it holds great opportunities in uncovering this patient's reorganization of cortical structures. The observation of individual functional connectivity could give deeper insight into maladaptive neuroplasticity and possibly its prevention via personalized rehabilitation therapy [34]. Not only patients with SCI could profit from a deeper investigation of functional connectivity analysis generally and during RHI. In patients with psychological disorders like schizophrenia or disturbed body perception, RHI can cause different responses [40, 41]. Looking closer at the effect of RHI in disorders and the correlation to connectivity patterns might become an interesting and maybe useful tool in clinical settings.

## 5. Limitations of the Study

Methodological limitations and technical restrictions of this study have to be considered when interpreting the results. First, the sample size of the group in whom the RHI could not be elicited is rather small, and the assessment of a single case of SCI is also not sufficient to draw definitive conclusions. The control group and the patient are not age matched which might have influenced the results. In SCI, the etiology varies a lot from patient to patient such that the sample would need to be very homogeneous—a general challenge to studies examining neuroplasticity in SCI. Due to the rather extensive study design and strict exclusion criteria, it was not possible in this case. Second, the relationship between TBS and the effectivity of the RHI can only be estimated according to behavioral data. Third, the data recorded during the RHI could not be compared with a resting condition. Finally, it cannot be excluded that HD-EEG is simply not suited to detect the fine-grained differences between brain activity in people who experience RHI and those who do not.

## 6. Conclusions

In this study, the patient with SCI showed a more intense phenomenon of lateralization than the healthy participants, which might be due to a higher effectivity of RHI in the patient and consequently to a neglect of the right hand. Still, the overall intensity of connectivity and the patterns do not seem to differ, pointing to an equalization of overall brain activity in the patient. We could not show any difference in connectivity between sham and real TBS, and the connectivity of the investigated brain areas does not seem to correlate with the behavioral report of whether the rubber hand is integrated into the bodily self-perception or not. This leads to the conclusion that the observed cortico-cortical communication is not influenced by—and does not influence—the effectivity of the RHI.

The results of this study shed new light on the processes taking place during visuo-sensory stimulation and raise further questions, such as how the illusion of integrating a fake body part is represented by brain activity, as assessed by using HD-EEG. Hence, it is still not clear to which extent bottom-up and top-down pathways contribute to the illusion. Further studies need to be conducted within larger samples of patients and healthy participants. One focus of future research could be functional connectivity patterns as biomarkers in patients with SCI, allowing for a better understanding of maladaptive neuroplasticity and individual rehabilitation approaches.

## Figures and Tables

**Table 1:** Global *p* values for the three comparisons for COH and ffDTF.

Global *p* values	COH	ffDTF
rTBS vs. sTBS	0.9072	0.9651
RHI vs. NoRHI with rTBS	0.7787	0.7010
RHI vs. NoRHI with sTBS	0.5601	0.7571

rTBS: real theta burst stimulation; sTBS: sham theta burst stimulation; RHI: rubber hand illusion; noRHI: no rubber hand illusion; COH: ordinary coherence; ffDTF: full frequency directed transfer function.

## Data Availability

The data used to support the findings of this study can be requested from the first author: v.frey@salk.at

## Supplementary Materials

Supplementary Materials S1 HD-EEG electrode setting and mapping of relevant brain areas. S2: Directed connectivity (ffDTF) after sham transcranial magnetic stimulation. The values show the percentile of the patient's data within the data of the control group (from 0 to 1). M1: primary motor cortex; SSC: primary somatosensory cortex; EBA: extrastriate body area; PMC: premotor cortex; IPS: intraparietal sulcus; r: right, l: left. S3: Directed connectivity (ffDTF) after real transcranial magnetic stimulation. The values show the percentile of the patient's data within the data of the control group (from 0 to 1). M1: primary motor cortex; SSC: primary somatosensory cortex; EBA: extrastriate body area; PMC: premotor cortex; IPS: intraparietal sulcus; r: right; l: left. S4: nondirected connectivity (COH) after real transcranial magnetic stimulation. The values show the percentile of the patient's data within the data of the control group (from 0 to 1). M1: primary motor cortex; SSC: primary somatosensory cortex; EBA: extrastriate body area; PMC: premotor cortex; IPS: intraparietal sulcus; r: right; l: left. S5: Nondirected connectivity (COH) after sham transcranial magnetic stimulation. The values show the percentile of the patient's data within the data of the control group (from 0 to 1). M1: primary motor cortex; SSC: primary somatosensory cortex; EBA: extrastriate body area; PMC: premotor cortex; IPS: intraparietal sulcus; r: right; l: left.
