# Supplementary material for: Connectivity Analysis during Rubber Hand Illusion—A Pilot TMS-EEG Study in a Patient with SCI
Source: Neural Plast. 2021 Feb 8;2021:6695530. doi: 10.1155/2021/6695530 (PMC7884106; doi:10.1155/2021/6695530)
Supplement: Supplementary Materials — S1 HD-EEG electrode setting and mapping of relevant brain areas. S2: Directed connectivity (ffDTF) after sham transcranial magnetic stimulation. The values show the percentile of the patient's data within the data of the control group (from 0 to 1). M1: primary motor cortex; SSC: primary somatosensory cortex; EBA: extrastriate body area; PMC: premotor cortex; IPS: intraparietal sulcus; r: right, l: left. S3: Directed connectivity (ffDTF) after real transcranial magnetic stimulation. The values show the percentile of the patient's data within the data of the control group (from 0 to 1). M1: primary motor cortex; SSC: primary somatosensory cortex; EBA: extrastriate body area; PMC: premotor cortex; IPS: intraparietal sulcus; r: right; l: left. S4: nondirected connectivity (COH) after real transcranial magnetic stimulation. The values show the percentile of the patient's data within the data of the control group (from 0 to 1). M1: primary motor cortex; SSC: primary somatosensory cortex; EBA: extrastriate body area; PMC: premotor cortex; IPS: intraparietal sulcus; r: right; l: left. S5: Nondirected connectivity (COH) after sham transcranial magnetic stimulation. The values show the percentile of the patient's data within the data of the control group (from 0 to 1). M1: primary motor cortex; SSC: primary somatosensory cortex; EBA: extrastriate body area; PMC: premotor cortex; IPS: intraparietal sulcus; r: right; l: left. [file 6695530.f1.pdf]

Supplementary:

S1: HD-EEG electrode setting and mapping of relevant brain areas.

S2: Directed connectivity (ffDTF) after sham transcranial magnetic stimulation. The values show the percentile of the patient's data within the data of the control group (from 0 to 1). M1= primary motor cortex, SSC= primary somatosensory cortex, EBA= extrastriate body area, PMC= premotor cortex, IPS= intraparietal sulcus, r= right, l= left.

S3: Directed connectivity (ffDTF) after real transcranial magnetic stimulation. The values show the percentile of the patient's data within the data of the control group (from 0 to 1). M1= primary motor cortex, SSC= primary somatosensory cortex, EBA= extrastriate body area, PMC= premotor cortex, IPS= intraparietal sulcus, r= right, l= left.

S4: Non-directed connectivity (COH) after real transcranial magnetic stimulation. The values show the percentile of the patient's data within the data of the control group (from 0 to 1). M1= primary motor cortex, SSC= primary somatosensory cortex, EBA= extrastriate body area, PMC= premotor cortex, IPS= intraparietal sulcus, r= right, l= left.

S5: Non-directed connectivity (COH) after sham transcranial magnetic stimulation. The values show the percentile of the patient's data within the data of the control group (from 0 to 1). M1= primary motor cortex, SSC= primary somatosensory cortex, EBA= extrastriate body area, PMC= premotor cortex, IPS= intraparietal sulcus, r= right, l= left.

# S2

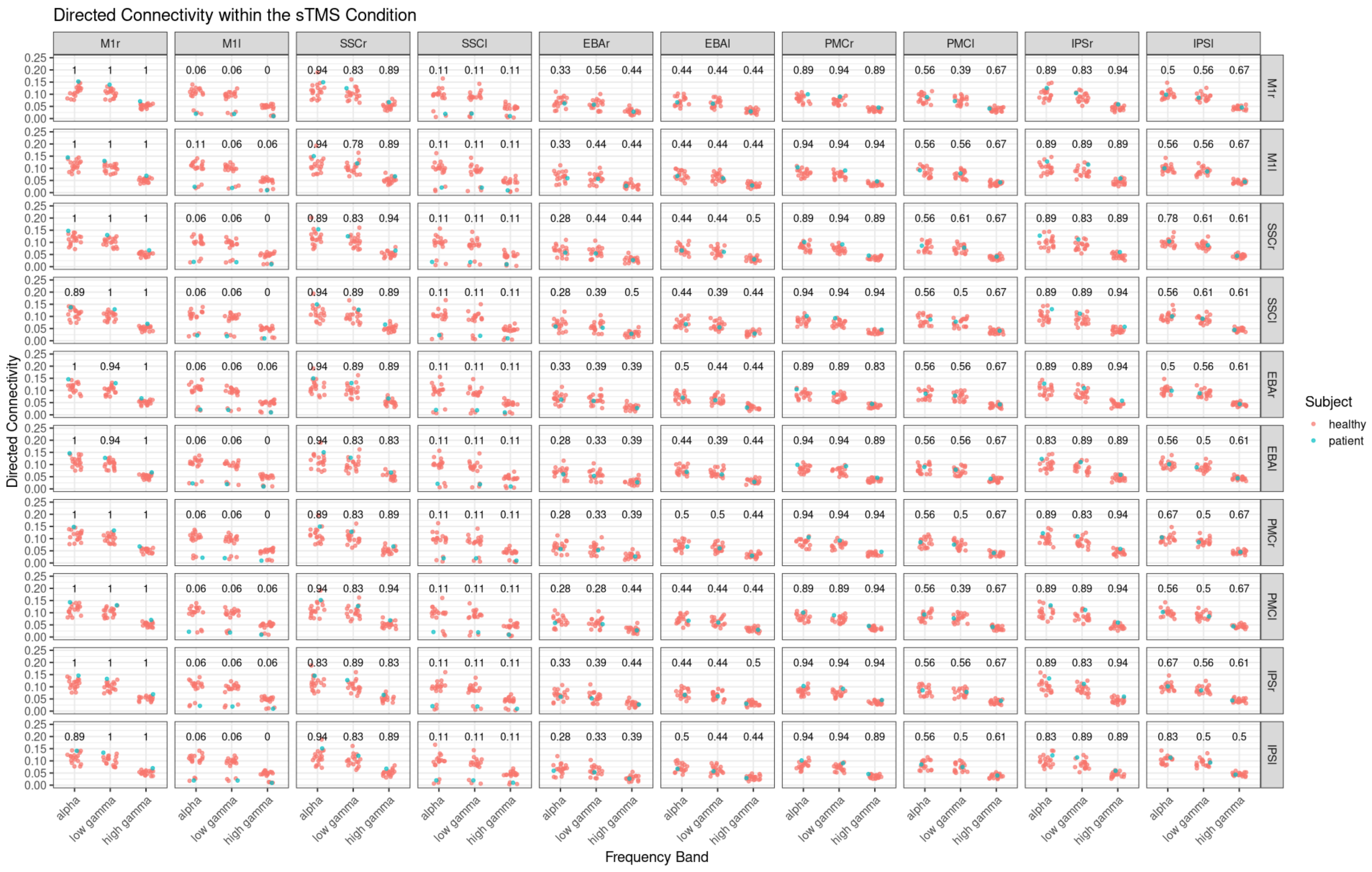


S2: Directed connectivity (ffDTF) after sham transcranial magnetic stimulation. The values show the percentile of the patients data within the data of the control group (from 0 to 1). M1= primary motor cortex, SSC= primary somatosensory cortex, EBA= extrastriate body area, PMC= premotor cortex, IPS= intraparietal sulcus, r= right, l= left.

# S3:

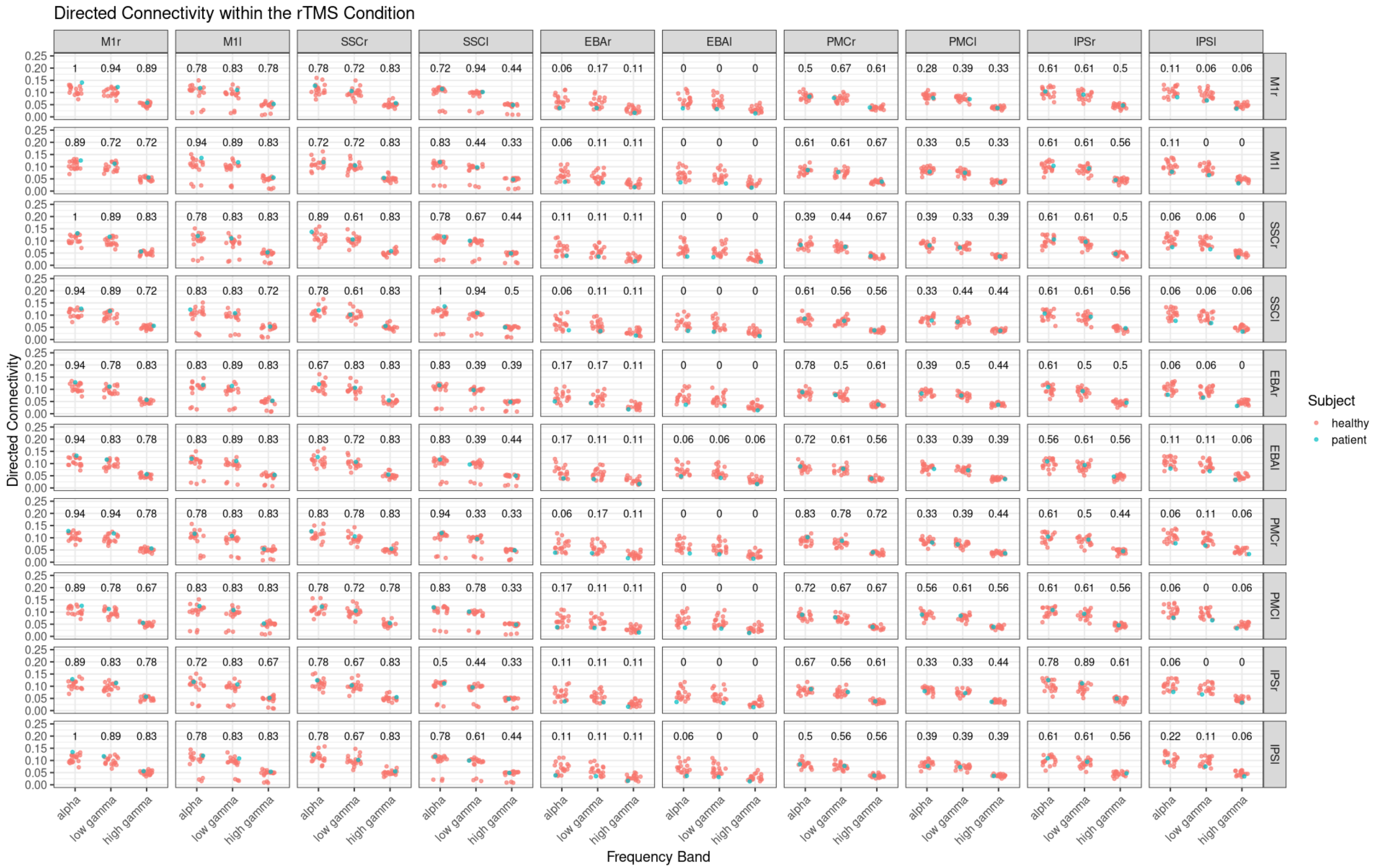


S3: Directed connectivity (ffDTF) after real transcranial magnetic stimulation. The values show the percentile of the patients data within the data of the control group (from 0 to 1). M1= primary motor cortex, SSC= primary somatosensory cortex, EBA= extrastriate body area, PMC= premotor cortex, IPS= intraparietal sulcus, r= right, l= left.

## S4:

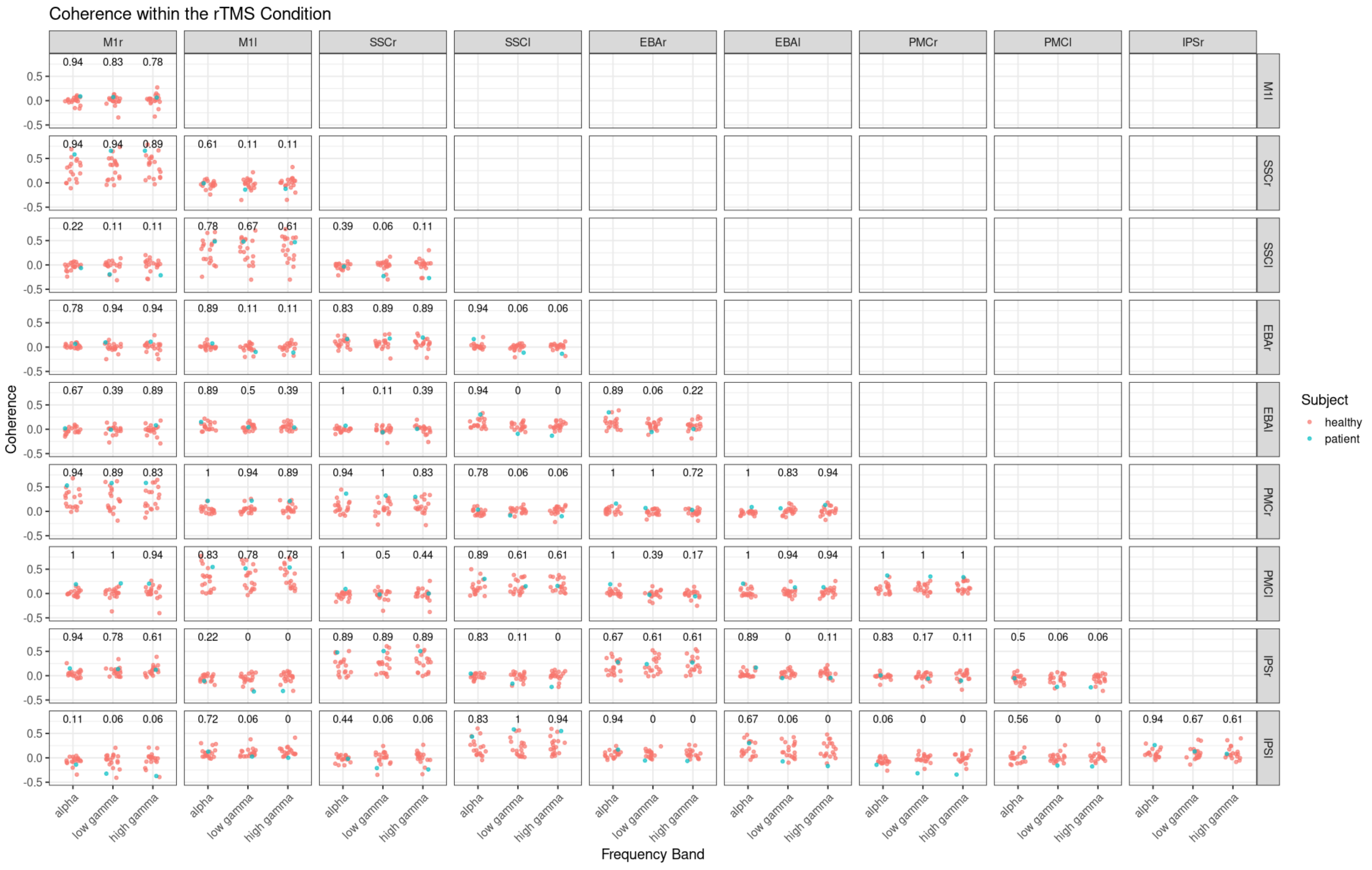


S4: Non-directed connectivity (COH) after real transcranial magnetic stimulation. The values show the percentile of the patients data within the data of the control group (from 0 to 1). M1= primary motor cortex, SSC= primary somatosensory cortex, EBA= extrastriate body area, PMC= premotor cortex, IPS= intraparietal sulcus, r= right, l= left.

**S5:**

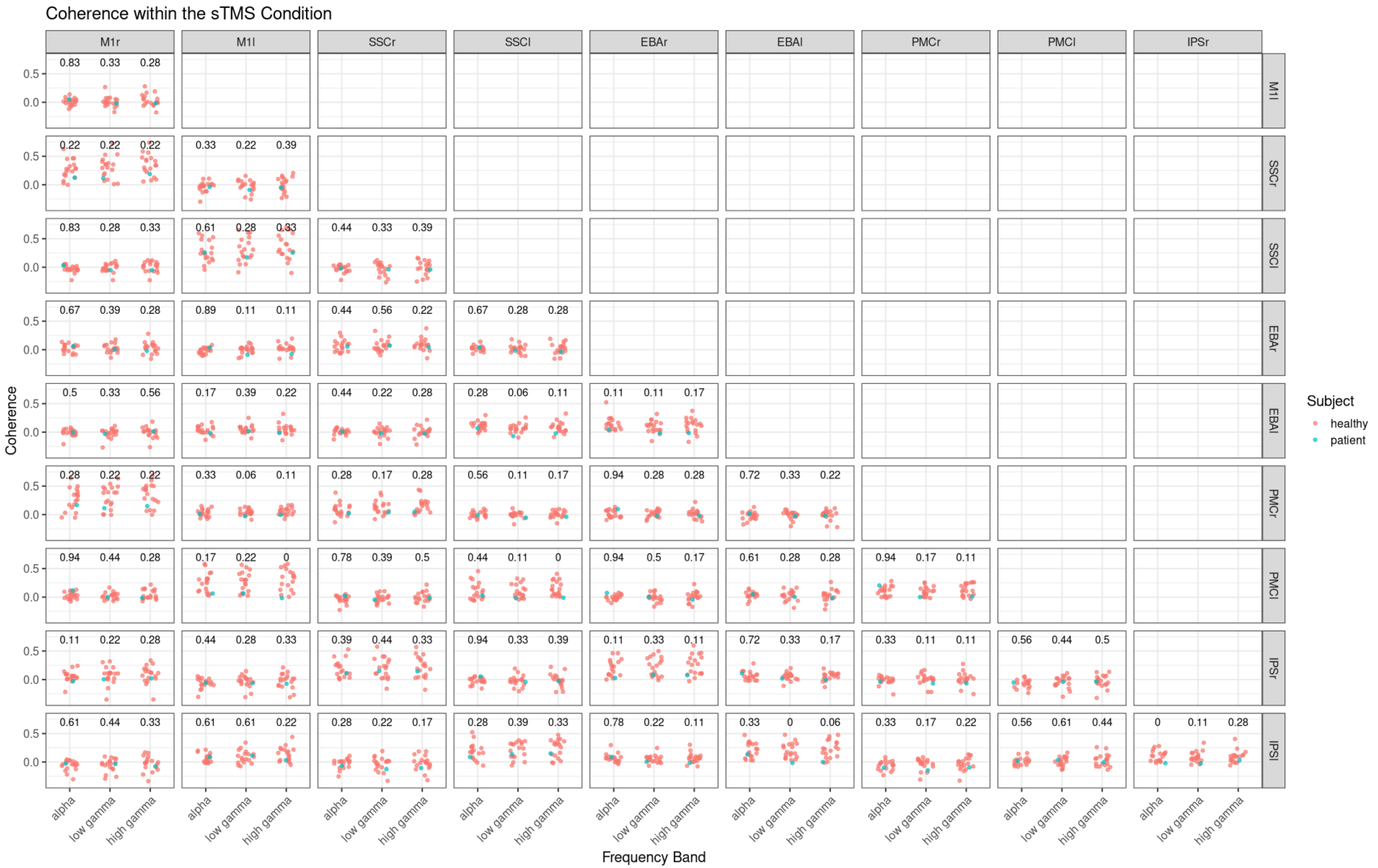


S5: Non-directed connectivity (COH) after sham transcranial magnetic stimulation. The values show the percentile of the patients data within the data of the control group (from 0 to 1). M1= primary motor cortex, SSC= primary somatosensory cortex, EBA= extrastriate body area, PMC= premotor cortex, IPS= intraparietal sulcus, r= right, l= left.
